# Low birth weight, preterm birth or small-for-gestational-age are not associated with dental caries in young Japanese children

**DOI:** 10.1186/1472-6831-14-38

**Published:** 2014-04-14

**Authors:** Keiko Tanaka, Yoshihiro Miyake

**Affiliations:** 1 Department of Preventive Medicine and Public Health, Faculty of Medicine, Fukuoka University, 814-0180 Fukuoka, Japan

**Keywords:** Child, Dental caries, Japan, Low birth weight, Preterm birth, Small-for-gestational-age

## Abstract

**Background:**

Low birth weight (LBW) continues to increase and is a major public health problem in Japan. In the present cross-sectional study, we examined the associations between LBW, preterm birth and small-for-gestational-age (SGA) and the prevalence of dental caries in young Japanese children.

**Methods:**

Study subjects were 2,055 children aged 3 years. Data on birth conditions were obtained through the transcription by parents or guardians of the information from their maternal and child health handbook, in which the data were recorded by staff at the birth hospital or clinic, to our self-administered questionnaire. Children were classified as having caries if one or more deciduous teeth were decayed, missing, or had been filled at the time of examination. Adjustments were made for sex, toothbrushing frequency, use of fluoride, regular dental check-ups, between-meal snack frequency, breastfeeding duration, paternal and maternal educational levels, maternal smoking during pregnancy, and secondhand smoke exposure at home.

**Results:**

The prevalence of dental caries was 20.7%. The mean birth weight was 3018.3 g, and 8.3% were classified as LBW (<2,500 g), 4.5% as preterm birth (<37 weeks), and 7.1% as SGA (<10th percentile). Preterm birth was associated with a 40% decreased prevalence of dental caries (adjusted prevalence ratio = 0.60, 95% confidence interval: 0.36–1.02, p = 0.06). There were no associations between LBW or SGA and the prevalence of dental caries.

**Conclusions:**

The results of the study failed to detect significant associations between LBW, preterm birth or SGA and the prevalence of dental caries in Japan. Further study is needed in other populations to confirm the generalizability of these findings.

## Background

According to the Vital Statistics of Japan, the rate of low birth weight (LBW) (<2,500 g) continues to increase, and in 2010, 9.7% of all live births in Japan were babies of LBW [1]. LBW is mostly found in cases of preterm birth. Children of preterm and LBW are prone to many serious medical problems, such as cerebral palsy, seizure disorders, severe mental retardation, and lower respiratory tract infections [2,3].

Preterm birth also deleteriously affects the structure of dental enamel [3], which is likely to increase susceptibility to dental caries. Nevertheless, epidemiological evidence on the relationship between preterm birth and/or LBW and dental caries has been limited, and the results have been inconsistent [4-7]. No association was observed between LBW and dental caries of the primary dentition among US children aged 2 to 6 years using cross-sectional data from the Third National Health and Nutrition Examination Survey [5], while preterm birth was significantly positively associated with the prevalence of dental caries in US children aged 3 to 5 years [7]. It is necessary to accumulate further evidence in order to clarify whether birth conditions affect dental caries in children. Investigations regarding the relationship between birth conditions and dental caries have been conducted in Western populations. To date, no epidemiological study has examined such relationships in non-Western populations. In the present cross-sectional study, we examined the associations between LBW, preterm birth and small-for-gestational-age (SGA) and the prevalence of dental caries in young Japanese children using data from the Fukuoka Child Health Study (FCHS).

## Methods

### Study population

The FCHS is a cross-sectional study to investigate the association between various selected factors and child health problems, such as dental caries and allergic disorders. In Japan, when children reach 3 years of age, the local municipality performs a physical examination, which includes an oral examination, anthropometric measurements of height and weight, and an interview with parents or guardians about the child’s health. Eligible subjects for the present study were those children aged 3 years who received the physical examination that was offered at all seven public health centers in Fukuoka City, a metropolitan area on Kyushu Island in southern Japan, with a total population of almost 1,414,000. During the period from June 2006 to January 2007, out of the 8,269 eligible children, the parents or guardians of 8,064 were provided with a self-administered questionnaire, a brief self-administered diet history questionnaire, and a postage-paid, addressed return envelope. The parents or guardians of 205 children declined the receipt of these materials or were failed to be provided with them. Ultimately, the parents or guardians of a total of 2,109 children answered the questionnaires and mailed these materials to the data management center (participation rate = 25.5%). Our research technicians completed missing answers and/or illogical data by telephone interview. The current study was restricted to subjects who provided complete information on the variables under study, leaving data on 2,055 children available for analysis (24.9% of all eligible children). Permission to perform this study was obtained from the ethics committee of the Faculty of Medicine, Fukuoka University.

### Measurements

At the time of the physical examination, the presence of dental caries was assessed by a visual examination without radiographs. Data on the dental examination were recorded at the tooth level by a dentist in a maternal and child health handbook, which had been provided by the municipality during the pregnancy and which included information on prenatal checkups as well as postnatal health conditions of both the mother and baby, and the growth of the child. Data on birth weight and gestational age at birth are also recorded by staff at the birth hospital or clinic in the maternal and child health handbook. In this study, parents or guardians of the children were required to transcribe the data on the dental examination, birth weight, and gestational age at birth from the maternal and child health handbook to our self-administered questionnaire. We classified children as having dental caries if one or more primary teeth had decayed, were missing, or had been filled. We defined LBW as a birth weight of less than 2500 g. Preterm birth was defined as a birth occurring at a gestational age of less than 37 weeks. SGA was defined as a birth weight below the 10th percentile of the Japanese neonatal anthropometric norms for babies of the same gestational age, gender, and parity published by Itabashi et al. in 2010 [8].

Information on sex, dental health behavior (such as toothbrushing frequency, use of fluoride, and pattern of dental care), between-meal snack habits, breastfeeding duration, paternal and maternal educational levels, maternal smoking during pregnancy, and secondhand smoke exposure at home were obtained from a structured self-administered questionnaire. Use of fluoride was defined as positive if children were reported to use fluoride agents, such as toothpaste and gel.

### Statistical analysis

Sex, toothbrushing frequency, use of fluoride, regular dental check-ups, between-meal snack frequency, breastfeeding duration, paternal and maternal educational levels, maternal smoking during pregnancy and secondhand smoke exposure at home were selected as a priori potential confounding factors. Prevalence ratios (PRs) and 95% confidence intervals (CIs) were estimated by binomial regression with the log link function [9-11]. All statistical analyses were performed using the SAS software package version 9.3 (SAS Institute, Inc., Cary, NC, USA).

## Results

Of the 2,055 children, 426 (20.7%) had experienced dental caries and 1,629 (79.3%) were caries free. The mean number of teeth with dental caries for all subjects and for subjects who had experienced caries was 0.70 and 3.4, respectively.

Characteristics of the study population are described in Table 1. About 40% of the subjects reported toothbrushing two or more times per day. Fluoride agents were used by approximately 85% of children. About 44% of children received regular dental check-ups. More than 41% of the subjects had between-meal snacks two or more times per day. About 45% of children were breastfed for less than 12 months. About 13% of children had mothers who had smoked during pregnancy, and 43.8% were exposed to secondhand smoke at home. The mean birth weight was 3018.3 g, and 8.3% were classified as LBW, 4.5% as preterm birth, and 7.1% as SGA.

**Table 1:** Distribution of selected characteristics in 2,055 children aged 3 years

**Variable**	**Overall**	**Low birth weight**	**Preterm birth**	**Small-for-gestational-age**
	**(n = 2055)**	**(n = 170)**	**(n = 93)**	**(n = 145)**
	***n*** **(%)**	***n*** **(%)**	***n*** **(%)**	***n*** **(%)**
Male sex	1086 (52.9)	92 (54.1)	61 (65.6)	79 (54.5)
Toothbrushing frequency (times/day)				
<2	1239 (60.3)	96 (56.5)	57 (61.3)	88 (60.7)
≥2	816 (39.7)	74 (43.5)	36 (38.7)	57 (39.3)
Use of fluoride	1738 (84.6)	137 (80.6)	75 (80.7)	115 (79.3)
Regular dental check-ups	895 (43.6)	64 (37.7)	32 (34.4)	56 (38.6)
Between-meal snack frequency (times/day)				
<1	444 (21.6)	33 (19.4)	10 (10.8)	35 (24.1)
1	751 (36.6)	75 (44.1)	37 (39.8)	61 (42.1)
≥2	860 (41.9)	62 (36.5)	46 (49.5)	49 (33.8)
Breastfeeding duration				
<12 months	916 (44.6)	97 (57.1)	56 (60.2)	83 (57.2)
≥12 months	1139 (55.4)	73 (42.9)	37 (39.8)	62 (42.8)
Paternal educational level (years)				
<13	568 (27.6)	53 (31.2)	33 (35.5)	43 (29.7)
13 − 14	308 (15.0)	24 (14.1)	19 (20.4)	16 (11.0)
≥15	1179 (57.4)	93 (54.7)	41 (44.1)	86 (59.3)
Maternal educational level (years)				
<13	581 (28.3)	55 (32.4)	22 (23.7)	49 (33.8)
13 − 14	822 (40.0)	60 (35.3)	38 (40.9)	60 (41.4)
≥15	652 (31.7)	55 (32.4)	33 (35.5)	36 (24.8)
Maternal smoking during pregnancy	271 (13.2)	25 (14.7)	12 (12.9)	25 (17.2)
Secondhand smoke exposure at home	899 (43.8)	67 (39.4)	41 (44.1)	59 (40.7)
Low birth weight^*^	170 (8.3)	–	–	–
Preterm birth^†^	93 (4.5)	–	–	–
Small-for-gestational-age^‡^	145 (7.1)	–	–	–

^*^Birth weight less than 2500 g.

^†^Birth occurring at a gestational age of less than 37 weeks.

^‡^Birth weight below the 10th percentile of the Japanese neonatal anthropometric norms for babies of the same gestational age, gender, and parity published by Itabashi et al. in 2010 [8].

Table 2 gives the PRs and their 95% CIs for dental caries in relation to LBW, preterm birth, and SGA. After adjustment for the confounding factors under study, preterm birth was associated with a 40% decreased prevalence of dental caries (adjusted PR = 0.60, 95% CI: 0.36-1.02, p = 0.06). There were no associations between LBW or SGA and the prevalence of dental caries.

**Table 2:** Prevalence ratios (PRs) and 95% confidence intervals (CIs) for dental caries in relation to birth conditions in 2055 Japanese children aged 3 years

	**Prevalence**	**Crude PR (95% CI)**	Adjusted PR^*^(95% CI)
Low birth weight			
No	395/1885 (21.0%)	1.00	1.00
Yes	31/170 (18.2%)	0.87 (0.63 − 1.21)	0.90 (0.66 − 1.24)
Preterm birth			
No	414/1962 (21.1%)	1.00	1.00
Yes	12/93 (12.9%)	0.61 (0.36 − 1.04)	0.60 (0.36 − 1.02)
Small-for-gestational-age			
No	398/1910 (20.8%)	1.00	1.00
Yes	28/145 (19.3%)	0.93 (0.66 − 1.31)	0.95 (0.68 − 1.33)

^*^Adjustment for sex, toothbrushing frequency, use of fluoride, regular dental check-ups, between-meal snack frequency, breastfeeding duration, paternal and maternal educational levels, maternal smoking during pregnancy, and secondhand smoke exposure at home.

Maternal smoking during pregnancy is the leading cause of LBW and preterm birth. It also might increase the risk of dental caries in children. In the present study, when children were classified according to maternal smoking status during pregnancy, an inverse association of preterm birth with the prevalence of dental caries was more pronounced in children whose mothers had never smoked during pregnancy (adjusted PR = 0.55, 95% CI: 0.29–1.01) than in those whose mothers had smoked during pregnancy (adjusted PR = 0.84, 95% CI: 0.33–2.15). No difference was observed in the association of preterm birth with the prevalence of dental caries between children with or without maternal smoking during pregnancy (p for homogeneity of PR = 0.42).

## Discussion

The present study found that preterm birth was associated with a 40% decreased prevalence of dental caries. Our findings are at variance with a previous cross-sectional study which showed that preterm birth was significantly positively associated with the prevalence of dental caries among US children aged 3 to 5 years [7]. On the other hand, our results showing no associations between LBW or SGA and dental caries are in agreement with those of other studies [4-7]. In a cross-sectional study in Brazilian children aged 6 years, a lack of association between LBW and a high level of dental caries (number of decay, missing and filled teeth ≥4) was observed [6]. According to a systematic review regarding the LBW and dental caries based on four studies in 2001, there was no evidence that LBW is a risk factor for caries in the primary dentition [2]. On the other hand, a prospective cohort study in the UK, in which birth weight was treated as a continuous variable across the whole range, showed that birth weight was positively associated with the risk of dental caries in 985 children aged 61 months: the adjusted OR per 100 g increase was 1.08 (95% CI: 1.03–1.13) [12]. Regarding SGA, a US cross-sectional study using data from the Third National Health and Nutritional Examination Survey showed that SGA was non-significantly inversely associated with dental caries in children aged 2 to 5 years [7]. However, it should be noted that the above mentioned studies used different definitions of outcome, study population, assessment methods, and confounding factors, thus limiting the feasibility of inter-study comparisons.

A longitudinal study among Australian children at 52 months of age showed that the prevalence of enamel defects, such as hypoplasia or opacity, was higher in preterm children than in full-term normal birth weight children [4]. It is speculated that these enamel defects predispose children to increased caries risk [13]. Nevertheless, the prevalence of dental caries in these two groups in the cited study was not significantly different [4]. Further, in a cross-sectional study among Brazilian children aged 0 to 3 years, the mean number of dental caries was higher in children born at full term than in those born prematurely [14]. Our observed marginally inverse association between preterm birth and dental caries may be explained by the fact that preterm birth children experienced more follow-up and had a greater opportunity to receive various forms of health information, including information on oral health, from the hospital than did full term birth children. Another possible explanation is that the delayed tooth eruption in children born preterm might contribute to a decreased risk of dental caries. In a retrospective study in Turkey, compared with children at >37 weeks of gestational age, children at ≤37 weeks of gestational age demonstrated a significant delayed eruption of the first primary tooth [15]. Alternatively, this association may be merely a coincidence.

No previous studies have addressed an interaction between birth conditions and prenatal smoking with respect to dental caries. Maternal smoking during pregnancy is the leading cause of LBW and preterm birth. Our previous study showed that prenatal smoke exposure was associated with an increased prevalence of dental caries in children [16]. Maternal smoking during pregnancy, LBW and preterm birth, and dental caries might be interrelated. In the current study, no interaction was observed between preterm birth and maternal smoking during pregnancy with respect to dental caries, however.

This research study had several methodological strengths. Study subjects were homogeneous in terms of age and geographic background. Information on birth weight and gestational age at birth was drawn from data recorded by hospital or clinic staff in the maternal and child health handbook. Data regarding dental caries were obtained from dental examinations by dentists. We were thus able to control for a variety of potential confounding factors.

Several methodological limitations of the current study should also be clarified. Of 8,269 eligible children in Fukuoka City, only 2055 (24.9%) were included in this analysis, leading to a potential for selection bias. The study subjects were probably not a representative sample of Japanese toddlers in the general population. In fact, the educational levels of the subjects’ parents in the present study were higher than those of the general population. According to the 2000 population census of Japan, the proportions of men 35 to 39 years of age in Fukuoka City with < 13, 13 to 14, ≥15, and unknown years of education were 39.6%, 8.0%, 43.3%, and 9.1%, respectively [17]. The corresponding figures among fathers in the present study were 27.6%, 15.0%, 57.4%, and 0.0%, respectively. The proportions of women 30 to 34 years of age in Fukuoka City with < 13, 13 to 14, ≥15, and unknown years of education were 41.3%, 34.4%, 16.1%, and 8.3%, respectively [17]. The corresponding figures among mothers in the present study were 28.3%, 40.0%, 31.7%, and 0.0%, respectively. On the other hand, the prevalence of dental caries in the study population (20.7%) appeared lower than that in a sample of 3-year-old Japanese children assessed in a 2005 survey of dental disease [18]. Further, it is difficult to generalize our findings to other populations because various factors, such as cultural and social factors and the prevalence of outcome, are different across populations. Although adjustments were made for several confounding factors, residual confounding effects could not be ruled out.

In this study, because the data on dental examinations were transcribed by parents or guardians of the children from their maternal and child health handbook to our self-administered questionnaire, we cannot exclude the possibility that transcription errors occurred. However, misclassification of outcome is unlikely to differ across categories of birth conditions. The non-differential outcome misclassification might have biased the magnitude of the observed associations toward the null.

## Conclusions

To our knowledge, there has been no prior study in Japan on the relationship between birth conditions and dental caries. The results of this cross-sectional study revealed no associations between LBW, preterm birth, or SGA and the prevalence of caries in primary dentition in Japanese children; however, there was a suggestion of an inverse association between preterm birth and caries (p = 0.06). Although the present study did not find an association between birth conditions and dental caries in the primary dentition, it is possible that such an association may exist in the permanent dentition. Further studies, particularly prospective studies, are needed in other populations to confirm that there is indeed no association between birth conditions and dental caries in children.

## Abbreviations

CI: Confidence interval; FCHS: Fukuoka Child Health Study; LBW: Low birth weight; PR: Prevalence ratio; SGA: Small-for-gestational-age.

## Competing interests

The authors declare that they have no competing interests.

## Authors’ contributions

Both authors contributed to the study concept and design and the acquisition of data. KT was responsible for the analysis and interpretation of data and the drafting of the manuscript. Both authors participated in critically revising the manuscript and approved the final version of the manuscript.

## Pre-publication history

The pre-publication history for this paper can be accessed here:

http://www.biomedcentral.com/1472-6831/14/38/prepub
